# Oncologic Outcomes of Patients with Immune Checkpoint Inhibitor Resistant Urothelial Carcinoma Treated with Enfortumab Vedotin and the Impact of Neutrophil-to-Lymphocyte Ratio and Dysgeusia on Overall Survival: A Retrospective Multicenter Cohort Study in Japan

**DOI:** 10.3390/cancers16152648

**Published:** 2024-07-25

**Authors:** Keita Nakane, Kazuki Taniguchi, Minori Nezasa, Torai Enomoto, Toyohiro Yamada, Risa Tomioka-Inagawa, Kojiro Niwa, Masayuki Tomioka, Takashi Ishida, Shingo Nagai, Shigeaki Yokoi, Tomoki Taniguchi, Makoto Kawase, Kota Kawase, Koji Iinuma, Yuki Tobisawa, Takuya Koie

**Affiliations:** 1 Department of Urology, Gifu University Graduate School of Medicine, Gifu 5011194, Japan; nakane.keita.k2@f.gifu-u.ac.jp (K.N.); enomoto.torai.d3@f.gifu-u.ac.jp (T.E.); tomioka.masayuki.p7@f.gifu-u.ac.jp (M.T.); taniguchi.tomoki.a8@f.gifu-u.ac.jp (T.T.); kawase.makoto.g5@f.gifu-u.ac.jp (M.K.); kawase.kota.b5@f.gifu-u.ac.jp (K.K.); iinuma.koji.s0@f.gifu-u.ac.jp (K.I.); tobisawa.yuki.a7@f.gifu-u.ac.jp (Y.T.); 2 Department of Urology, Gifu Prefectural General Medical Center, Gifu 5008717, Japan; glay.apologize@gmail.com; 3 Department of Urology, Matsunami General Hospital, Gifu 5016062, Japan; nezasa001@gmail.com; 4 Department of Urology, Ogaki Municipal Hospital, Ogaki 5038502, Japan; to.yama0212@gmail.com; 5 Department of Urology, Japanese Red Cross Takayama Hospital, Takayama 5068550, Japan; s11110032@gmail.com; 6 Department of Urology, Daiyukai Daiichi Hospital, Ichinomiya 4918551, Japan; kniwa2004813@gmail.com; 7 Department of Urology, Chuno Kosei Hospital, Seki 5013802, Japan; 8 Department of Urology, Gifu Municipal Hospital, Gifu 5008513, Japan; justaskaxis@gmail.com; 9 Department of Urology, Toyota Memorial Hospital, Toyota 4718513, Japan; shingo-nagai@nifty.com; 10 Department of Urology, Central Japan International Medical Center, Minokamo 5058510, Japan; s-yokoi@cjimc-hp.jp

**Keywords:** retrospective multicenter cohort study, advanced urothelial carcinoma, enfortumab vedotin, overall survival, prognostic factor, neutrophil-to-lymphocyte ratio, dysgeusia

## Abstract

**Simple Summary:**

Patients with locally advanced or metastatic urothelial carcinoma have a poor prognosis. Enfortumab vedotin, administered after cisplatin-based chemotherapy and followed by immune checkpoint inhibitors, is widely known to prolong overall survival (OS). However, the predictive factors of enfortumab vedotin treatment that prolong OS remain unclear. In this study, patients who received enfortumab vedotin with shrinking tumors showed a significant increase in OS compared to those who received chemotherapy other than enfortumab vedotin or those who did not receive any treatment. Multivariate analysis identified neutrophil-to-lymphocyte ratio and dysgeusia as potential predictors of OS. Patients without these factors had a significantly prolonged OS compared to those with both factors. In real-world practice, enfortumab vedotin therapy is effective for patients with locally advanced or metastatic urothelial carcinoma.

**Abstract:**

Randomized phase III trial results have demonstrated enfortumab vedotin (EV), an antibody–drug conjugate (ADC) consisting of an anti-Nectin-4 human IgG1 monoclonal antibody and monomethyl auristatin E, is a useful treatment for patients with locally advanced or metastatic urothelial carcinoma (la/mUC) that progressed after immune checkpoint inhibitor (ICI) therapies. This multicenter retrospective cohort study aimed to identify predictive factors for the efficacy of EV therapy and prolonged overall survival (OS) of patients in clinical practice. This study included patients with la/mUC who received ICI treatment. Patients who subsequently received EV treatment, those who received non-EV chemotherapy, and those who received no treatment were defined as EV, non-EV, and best supportive care (BSC) groups, respectively. The median OS was 20, 15, and 7 months in the EV, non-EV, and BSC groups, respectively (*p* < 0.001). Patients with la/mUC who had a complete or partial response after EV treatment had a significantly prolonged OS compared with those with stable or progressive disease. Univariate analysis showed age, neutrophil-to-lymphocyte ratio (NLR), dysgeusia, and rash as independent predictors of OS improvement. NLR and dysgeusia were independent predictors of OS after EV in multivariate analysis. Patients without these factors had a significantly prolonged OS compared to those with both factors. In real-world practice, EV therapy is an effective treatment for patients with la/mUC after ICI treatment.

## 1. Introduction

In the United States, an estimated 169,360 new cases and 32,350 deaths occur annually due to urinary tract-associated malignant tumors [1]. Of these, 83,190 new cases and 16,840 deaths were attributed to bladder cancer, the most frequent malignant neoplasm among urothelial carcinomas (UCs) [1]. Approximately 5% of patients with bladder cancer are estimated to have regional or distant metastases at the time of diagnosis [1]. Furthermore, approximately 10–30% of patients who undergo radical cystectomy with urinary diversion develop local recurrence or distant metastases postoperatively [2,3]. In patients with UC, including upper urinary tract and bladder cancers, the prognosis for metastatic or unresectable cases is extremely poor [4,5]. Platinum-based chemotherapy is currently the standard first-line treatment for locally advanced or metastatic UC (la/mUC) [6,7]. Therefore, several guidelines recommend platinum-based combination chemotherapy as the first-line therapy and immune checkpoint inhibitors (ICIs) targeting programmed cell death 1 (PD-1) or programmed cell death ligand 1 (PD-L1) as the second-line therapy for the treatment of la/mUC [3,8,9]. Nivolumab, an anti-PD-1 antibody, is a useful adjuvant therapy for patients who have undergone radical cystectomy after neoadjuvant cisplatin-based chemotherapy and have residual UC in the muscle layer or lymph node metastases [10]. In addition, avelumab, an anti-PD-L1 antibody, has been shown to be useful as a maintenance therapy for patients who show no progression after platinum-based chemotherapy for la/mUC [11]. Enfortumab vedotin (EV) is widely recognized as an effective treatment for patients with la/mUC that progressed after ICI treatment [12]. EV is an antibody–drug conjugate (ADC) consisting of an anti-Nectin-4 human IgG1 monoclonal antibody and monomethyl auristatin E (MMAE) with microtubule polymerization inhibitory activity covalently conjugated via a linker [12,13]. EV is taken up by the body after binding to Nectin-4, which is a Ca^2+^-independent immunoglobulin-like protein that is highly expressed on the surface of cancer cells; subsequently, MMAE is released into the cells to inhibit cell division, induce apoptosis, and exert its anti-tumor effect [12,13]. In 2024, the EV302 trial investigating the efficacy of pembrolizumab plus EV as a first-line therapy for untreated la/mUC reported a significant improvement in overall survival (OS) compared to conventional platinum-based chemotherapy [14]. Therefore, a combination therapy consisting of EV and pembrolizumab is expected to become the first-line therapy for la/mUC treatment in the future [14].

In clinical practice, the number of patients who can receive EV after ICI treatment is not very large, and a treatment strategy that allows the prompt administration of sequential treatment is necessary [5]. In addition, factors that predict the effectiveness of EV therapy remain unknown. Therefore, this multicenter, retrospective cohort study aimed to examine the efficacy of EV after pembrolizumab treatment in the real world and identify factors that predict prognosis in patients with la/mUC who received EV therapy.

## 2. Materials and Methods

### 2.1. Patients

This study was approved by the Institutional Review Board of Gifu University (approval number: 2023-144). Owing to the retrospective nature of the study, informed consent was not acquired from each eligible patient, and an opt-out was used alternatively. In accordance with the provisions of ethics committees and ethical guidelines in Japan, retrospective and cohort studies using existing documents and other materials do not require written consent since the research information is disclosed to the public. Details of this retrospective cohort study, available only in Japanese, can be accessed at the following URL: https://rinri.med.gifu-u.ac.jp/esct/publish_document.aspx?ID=2842 (accessed on 1 June 2024).

This retrospective multicenter cohort study included patients with la/mUC who received ICIs such as nivolumab or pembrolizumab at 10 Japanese institutions between December 2017 and May 2023. All enrolled patients received platinum-based anticancer therapy and ICI therapy. Patients who were not evaluated for treatment response assessment after EV administration, those with missing data, and those who did not develop la/mUC progression after ICI treatment were excluded. Patient baseline information was obtained through anamnesis, physical examinations, and computed tomography (CT) scans of the chest-to-pelvic region. Clinical, laboratory, and tumor-related covariates were investigated, including age, sex, height, weight, body mass index, Eastern Cooperative Oncology Group Performance Status (ECOG-PS) [15], smoking history, primary tumor site, metastatic site, presence or absence of treatment for the primary tumor, hemoglobin (Hb) level, serum albumin (Alb) level, c-reactive protein (CRP), neutrophil count, lymphocyte count, neutrophil-to-lymphocyte ratio (NLR), presence of any neoadjuvant/adjuvant therapy, history of systemic therapy prior to ICI therapy (excluding neoadjuvant and adjuvant therapy), and treatment-related adverse events (AEs), including anemia, dysgeusia, peripheral neuropathy, fatigue, nausea, neutropenia, and rash. The severity of treatment-related AEs was assessed using the National Cancer Institute Common Terminology Criteria for AEs (CTCAE) version 5.0 [16]. Tumor staging for all patients was determined according to the 8th edition of the American Joint Committee on Cancer Staging Manual [17].

### 2.2. Treatment Schedule of EV Therapy

Patients who received EV therapy were treated with the same treatment schedule as that used in the EV301 study [12], namely, one cycle of 4 weeks, with EV administered at a dose of 1.25 mg/kg on days 1, 8, and 15. EV therapy was continued until imaging studies confirmed disease progression (PD), treatment refusal by the patient, or EV treatment was deemed intolerable due to advanced treatment-related AEs according to CTCAE version 5.0 [16].

### 2.3. Patient Evaluation

Imaging studies were scheduled at the discretion of each physician and continued until PD was confirmed. The best overall response after EV therapy was assessed using the Response Evaluation Criteria in Solid Tumors guidelines version 1.1 [18] and defined as complete response (CR), partial response (PR), stable disease (SD), or PD. The cutoff value of the clinical covariate based on the area under the receiver operating characteristic (ROC) curve (AUC) was defined as the minimum value of (1 − sensitivity)^2^ + (1 − specificity)^2^ [19].

### 2.4. Endpoints and Statistical Analysis

The primary endpoint of this study was to investigate the predictive factors for prolonged OS after EV treatment initiation. All enrolled patients were divided into three groups, including those who received EV as a third-line therapy (EV group), those who received non-EV chemotherapy (non-EV group), and those who were unable to continue treatment for any reason (best supportive care [BSC] group). OS was evaluated in the three groups, starting at the beginning of ICI and tertiary treatments, and the efficacy of EV was examined compared with other treatments. A log-rank test for clinical parameters was used to identify predictors of all-cause mortality after the initiation of EV treatment. In addition, univariate and multivariate analyses were performed using the Cox proportional hazards model to identify factors associated with mortality after EV treatment. Regarding the Cox proportional hazards model, the determination of cutoff values was based on the AUCs. The Kruskal–Wallis test was used for continuous variables, whereas Fisher’s exact test was used for categorical variables. All statistical analyses were performed using EZR version 1.56 (Saitama Medical Center, Jichi Medical University, Saitama, Japan), a graphical user interface of R version 3.3.0 (The R Foundation for Statistical Computing, Vienna, Austria) [20].

## 3. Results

### 3.1. Patient Characteristics

**Figure 1:**
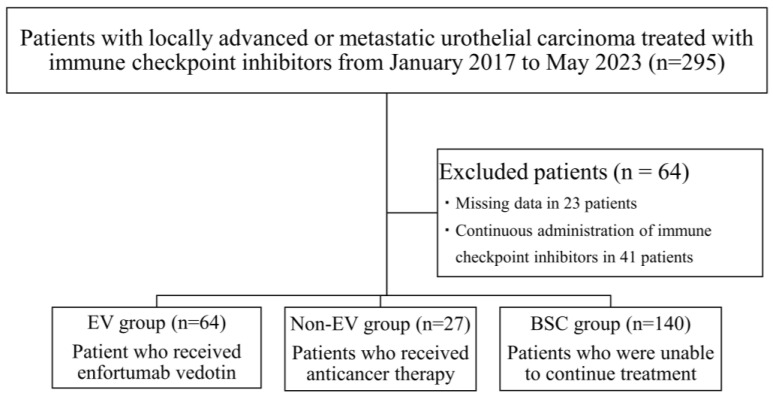
Flow diagram of the patient selection process.

Between December 2017 and May 2023, 295 patients with chemotherapy-refractory la/UC were treated with ICI, including adjuvant and maintenance therapies, at 10 Japanese institutions. Among them, 23 patients with missing data and 41 patients who received continuous ICI treatment due to a lack of PD were excluded from the analysis. Finally, 231 patients were enrolled in the final analysis. Figure 1 shows the number of patients in each group.

Table 1 shows the patient backgrounds and clinical and pathological data. For all patients, the median age at the start of ICI treatment was 72 years; similarly, the median age at the start of third-line treatment was 72 years. ECOG-PS was ≥1 in 67.5% of patients, 54.5% had bladder cancer as the primary tumor, and 77.5% had pure UC (PUC). Defensive therapy was performed at the primary site in 68.9% of the patients and in 67.4% of those who received neoadjuvant or adjuvant therapy. The median follow-up period was 8.0 months from the initiation of ICI treatment and 6.0 months from the initiation of the third-line treatment. The duration of ICI treatment was significantly longer in the third-line treatment groups than in the BSC group. Maintenance therapy with avelumab and adjuvant nivolumab was administered to 7.8% of patients, and these treatments were administered significantly more to patients in the EV group compared to those in the other groups (*p* = 0.007 and *p* < 0.001, respectively). Of the 27 patients in the non-EV group, 16 patients received gemcitabine and paclitaxel, 3 received gemcitabine and cisplatin, and 3 received gemcitabine and carboplatin. Additionally, one case each received irinotecan and cisplatin, docetaxel only, paclitaxel only, tegafur/uracil only, and gemcitabine only. The median Alb, neutrophil, lymphocyte, Hb levels, and NLR for all patients were 3.6 g/dL, 4395/µL, 1038/µL, and 10.8 g/dL, respectively. ECOG-PS and Alb, neutrophil, lymphocyte, and Hb levels were not available for the BSC group since data were collected only at the start of the ICI treatment.

### 3.2. Oncological Outcomes

**Figure 2:**
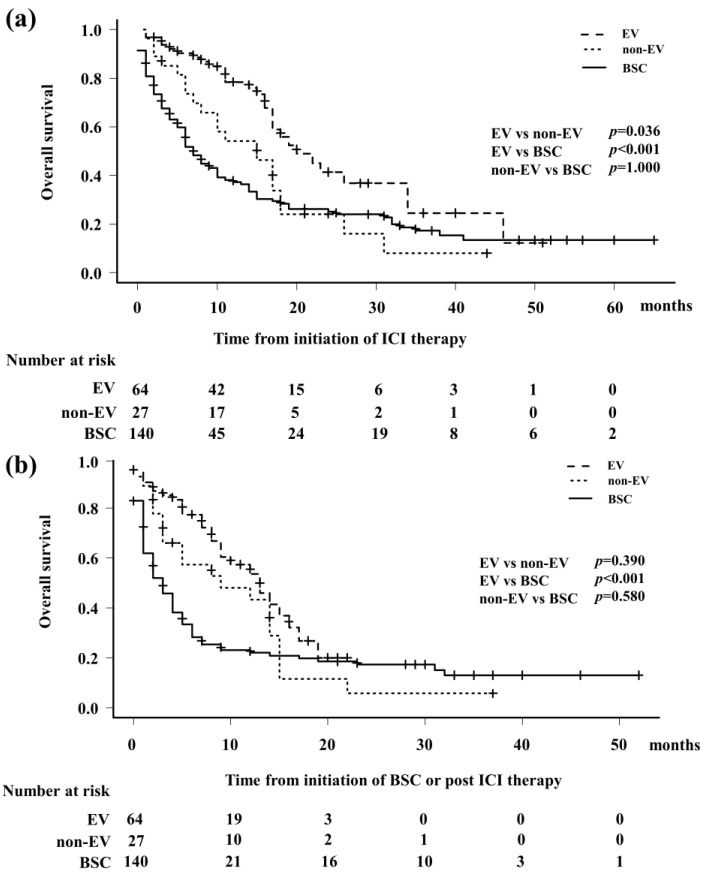
Kaplan–Meier analysis was used to compare the overall survival (OS) of the enfortumab vedotin (EV) group (patients treated with EV), non-EV group (patients treated with anticancer therapy other than EV), and BSC group (patients who were unable to continue treatment. (**a**) Median OS from the initiation of immune checkpoint inhibitor therapy was 20 months (95% confidence interval [CI]: 17–34 months), 15 months (95% CI: 7–18 months), and 7 months (95% CI: 6–10 months) in the EV, non-EV, and BSC groups, respectively (*p* < 0.001). (**b**) Median OS from the initiation of the third-line therapy was 13 months (95% CI: 9–16 months), 9 months (95% CI: 3–14 months), and 3 months (95% CI: 2–4 months) in the EV, non-EV, and BSC groups, respectively (*p* < 0.001).

At the end of the follow-up period, 150 (64.9%) patients had died, of whom 144 (62.3%) died from cancer-related causes, 1 (0.4%) from aspiration pneumonia, 1 (0.4%) from infection, and 4 (1.7%) from unknown causes. The median OS from ICI initiation in the EV, non-EV, and BSC groups was 20 (95% confidence interval [CI]: 17–34 months), 15 (95% CI: 7–18 months), and 7 months (95% CI: 6–10 months), respectively (Figure 2a). The 1-year OS rate from the initiation of ICI treatment was 78.4% (95% CI: 64.9–87.2%), 37.3% (95% CI: 28.7–45.8%), and 17.3% (95% CI: 10.4–25.6%) in the EV, non-EV, and BCS groups, respectively (Figure 2a). The median OSs from the start of treatment in the EV, non-EV, and BSC groups were 13 months (95% CI: 9–16 months), 9 months (95% CI: 3–14 months), and 3 months (95% CI: 2–4 months), respectively (Figure 2b). The 1-year OS rates from the initiation of EV were 53.8% (95% Cl: 36.9–68.0%), 43.3% (95% CI: 23.2–61.8%), and 22.1% (95% CI: 14.8–30.4%) in the EV, non-EV, and BSC groups, respectively.

**Figure 3:**
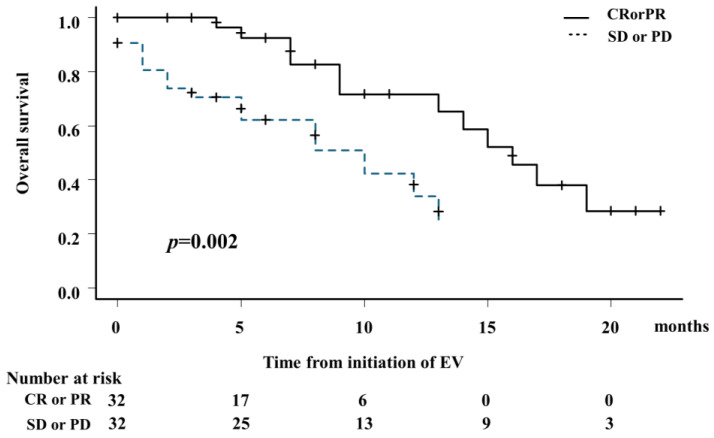
Kaplan–Meier analysis of OS by treatment response to EV. Median OS for patients with complete or partial response and stable or progressive disease after EV administration was 16 months (95% CI, 9–not applicable [NA]) and 10 months (95% CI: 5–13 months), respectively (*p* = 0.002).

In evaluating OS by treatment effect after EV administration, the median OS was 16 months (95% CI: 9–not applicable [NA]) for patients who achieved CR or PR and 10 months (95% CI: 5–13 months) for those who had SD or PD (*p* = 0.002; Figure 3). The 1-year survival rate was 71.7% (95% CI: 46.9–86.4%) for patients who obtained CR or PR and 33.9% (95% CI: 12.8–56.6%) for those who had SD or PD (Figure 3).

**Figure 4:**
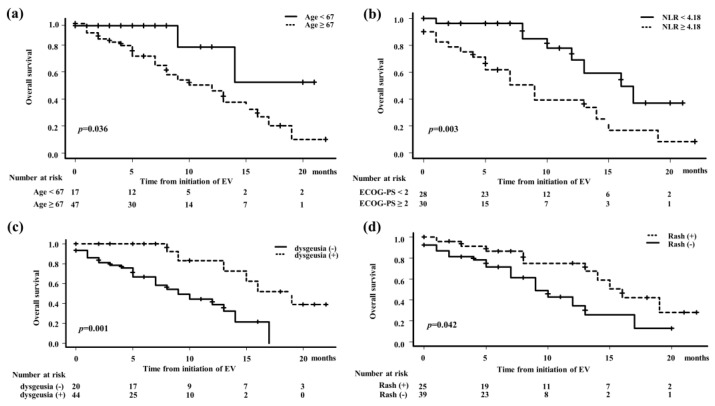
Kaplan–Meier analysis was used to compare OS in various covariables. (**a**) Median OS for patients aged ≥67 years and <67 years was 16 months (95% CI: 12–NA) and 9 months (95% CI: 5–14 months), respectively. (**b**) Median OS for patients with NLR <4.18 and NLR ≥4.18 was not reached (95% CI: 9–NA) and 12 months (95% CI: 7–16 months), respectively. (**c**) Median OS for patients with or without dysgeusia was 19 months (95% CI: 9–NA) and 0 months (95% CI: 5–14), respectively. (**d**) Median OS for patients with or without a rash was 16 months (95% CI: 8–NA) and 9 months (95% CI: 7–12 months), respectively.

For subgroup analysis of OS, patients were divided into two groups based on ROC curves for age at the initiation of EV treatment (≥67 vs. <67 years) and NLR (≥4.18 vs. <4.18); the presence or absence of EV-related AEs such as dysgeusia and rash were also compared. A significantly longer OS was observed in patients with an age ≥67 years, NLR <4.18, dysgeusia, and rash (Figure 4).

Table 2 enumerates the treatment-related AEs after EV administration in the EV group. Although Grade ≥3 anemia, decreased neutrophil counts, and rash were observed, all of the patients’ symptoms improved with EV withdrawal.

Univariate and multivariate Cox proportional hazards model analyses were performed to investigate variables associated with OS (Table 3). In the univariate analysis, NLR, dysgeusia, and rash were predictors associated with OS, whereas multivariate analysis showed that NLR and dysgeusia were independent predictors associated with OS.

**Figure 5:**
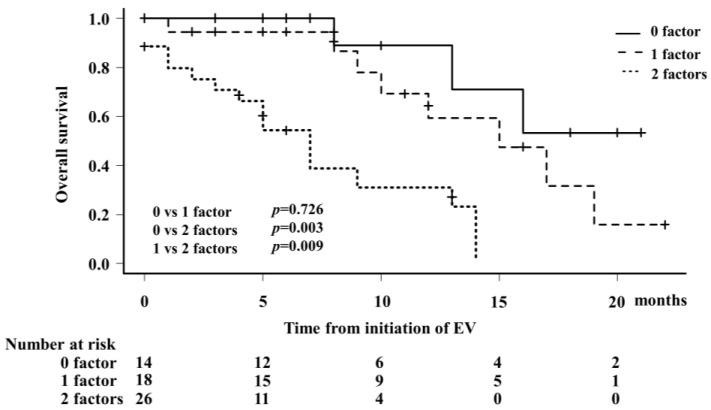
Kaplan–Meier analysis was performed in the EV group for patients who had neither of the two factors, namely neutrophil-to-lymphocyte ratio ≥4.18 and positive dysgeusia, as the 0 factor group, those who had either of the two factors as the 1 factor group, and those who had both factors as the 2 factor group. The median OS from the date of EV administration in the 0, 1, and 2 factor groups was NA (95% CI: 8–NA), 15 months (95% CI: 9–19 months), and 7 months (95% CI: 3–13 months), respectively.

Using Kaplan–Meier curve analysis to evaluate OS, patients treated with EV were divided into three groups based on the total points of NLR ≥ 4.18 and positive dysgeusia as one factor each (Figure 5). The median OS from the date of EV initiation was NA (95% CI: 8–NA), 15 months (95% CI: 9–19 months), and 7 months (95% CI: 3–13 months) for patients with zero, one, and two factors, respectively. The 1-year OS rate was 88.9% (95% CI: 43.3–98.4%) in patients without any factor, 59.4% (95% CI: 27.1–81.2%) in those with one factor, and 31.0% (95% CI: 10.7–54.2%) in those with two factors.

## 4. Discussion

Patients with la/mUC are known to have unfavorable prognoses, and there is a need to establish useful sequential therapies to prolong OS and identify predictive parameters for therapeutic efficacy in these patients [4,5,21,22]. Currently, platinum-based chemotherapy is the standard treatment for patients with la/mUC [3,6,7], and the usefulness of ICI therapies as a second-line treatment has been reported [4,8]. Pembrolizumab is recommended as a second-line therapy for patients with disease progression after the first-line therapy or for those who relapsed within a short duration after neoadjuvant chemotherapy followed by definitive therapies, including radical cystectomy or radiation for the primary sites [3,4,8]. In a phase III randomized controlled KEYNOTE 045 trial, pembrolizumab significantly prolonged OS compared with other chemotherapies in patients with la/mUC who were refractory to platinum-based chemotherapy [8]. The median OS in the KEYNOTE 045 trial was 10.3 months (95% CI: 8.0–11.8) in the pembrolizumab group compared with 7.4 months (95% CI: 6.1–8.3) in the other chemotherapy group (hazard ratio [HR] for death: 0.73; 95% CI: 0.59–0.91; *p* = 0.002) [8]. Thus, only a limited number of patients receive subsequent treatment in real-world clinical practice, even though the combination of cisplatin-based anticancer therapy and ICI therapy may have some advantages in improving oncological outcomes, such as OS [5].

EV is an ADC consisting of an anti-nectin-4 human IgG1 monoclonal antibody covalently linked to MMAE that exerts its anti-tumor effect by binding to a nectin-4 antibody and being internalized by cells [12,13]. The open-label phase III EV301 trial in patients with la/mUC who received platinum-based chemotherapy and whose disease progressed during or after treatment with ICIs showed improved oncological outcomes with EV [12]. The median OS was 12.88 months in the EV group and 8.97 months in the non-EVchemotherapy group, with an HR for OS of 0.70 (95% CI: 0.56–0.89), indicating that treatment with EV significantly prolonged OS in patients with la/mUC (*p* = 0.001) [12]. In addition, the results of the EV302 study, which investigated the efficacy of pembrolizumab plus EV as a first-line therapy for untreated la/mUC, have been published [14]. The median progression-free survival (PFS) was 12.5 months in the EV–pembrolizumab group and 6.3 months in the non-EV chemotherapy group, showing significantly longer PFS (*p* < 0.001), with an HR of 0.45 (95% CI: 0.38–0.54) for PD or death [14]. The median OS was also 31.5 months in the EV–pembrolizumab group and 16.1 months in the non-EV chemotherapy group, with similar results as those for PFS (HR for death: 0.47; 95% CI: 0.38–0.58; *p* < 0.001) [14]. Based on these findings, combination therapy consisting of EV and ICI is expected to have a high potential to improve the oncologic outcome of la/mUC [14]. Although several reports have predicted the therapeutic efficacy of EV, no useful factors that can accurately predict the efficacy of EV therapy have been identified [23,24,25,26,27].

In this study, we identified NLR and negative dysgeusia as useful prognostic factors in patients with la/mUC after EV administration. This suggests that the development of EV-related AEs may correlate with the response of patients with la/mUC to EV treatment. The NLR may reflect inflammation caused by urothelial carcinoma as well as other carcinomas [28,29,30], suggesting that patients with a lower inflammatory response may be more likely to have less aggressive cancer progression and thus may be more effectively treated with EVs. In a retrospective analysis of 109 patients with metastatic UC (mUC) who received EV, the CRP–albumin ratio (CAR), NLR, platelet–lymphocyte ratio, and lactate dehydrogenase were examined as predictors of treatment response after EV administration [31]. In a receiver operating characteristic curve analysis for predicting the treatment effect of EV, CAR was identified as a significant marker compared to other parameters [32]. Logistic regression analysis also identified ECOG-PS ≥ 1 (*p* = 0.04) and CAR ≥ 1 (*p* < 0.001) as independent predictors of a treatment effect for EV [32]. In a multicenter retrospective multicenter cohort study of 100 patients with mUC who received pembrolizumab or avelumab followed by EV, multivariate analysis revealed histological variant (*p* < 0.001), liver metastasis (*p* = 0.002), low serum albumin level (*p* = 0.003), and high CRP level (*p* = 0.011) were significantly associated with a poorer response to treatment of EV after ICI administration [31]. However, there have been no reports to date that NLR is a useful predictor of treatment effect after EV administration in patients with mUC.

In a study investigating the expression of Nectin-4 in normal human tissues and various cancer tissues, immunohistochemical analysis of 294 normal tissue specimens representing 36 human organs showed homogeneous weak-to-moderate staining mainly in human skin keratinocytes, skin appendages (sweat glands and hair follicles), transitional epithelium of the bladder, salivary glands (ducts), esophagus, breast, and stomach tissue [26]. Although the distribution of taste cells, nerves, and Nectin-4 in tongue tissue is unknown, Miyake et al. compared the incidence of dysgeusia between EV and other anticancer agents and showed that dysgeusia occurs more specifically with EV [27]. In addition, a longitudinal questionnaire survey using the chemotherapy-induced taste alteration scale (CiTAS) has been conducted in patients with la/mUC treated with systemic chemotherapy and/or immunotherapy [27]. Although platinum-based chemotherapy and treatment with ICIs did not significantly change the CiTAS, EV therapy induced significant dysgeusia, leading to the conclusion that EV therapy for la/mUC may induce severe dysgeusia that is not usually observed with other systemic therapies [27]. The relationship between Nectin-4 expression and EV efficacy was examined using Nectin-4-specific fluorescence in in situ hybridization to investigate the hypothesis that EV has a high affinity for Nectin-4 and, therefore, has higher efficacy in tumors with high Nectin-4 expression [23]. Compared to only 32% (24 of 74) in the non-amplified subgroup, 96% (27 of 28) of patients with Nectin-4 amplification had an objective response to EV (*p* < 0.001) [23]. A multivariate Cox analysis adjusted for age, sex, and Bellmunt risk factors reported that Nectin-4 amplification reduced the risk of death by 92% (95% CI: 0.02–0.34; *p* < 0.001) [23]. Using next-generation sequencing data from patients with la/mUC receiving EV treatment to investigate the relationship between the genetic characteristics of tumor tissue and clinical outcomes, patients with abundant mutations in *TP53*, *KDM6A*, and *MDM2* showed a better response to EV treatment [24]. Patients with these changes, as well as combined *TP53*/*MDM2* mutations, had better observed response rates (ORRs) with EV treatment than patients without these changes [24]. In univariate analysis, a baseline Alb level ≥3.0 g/dL and the presence of *TP53*/*MDM2* composite change were associated with longer OS, and baseline ECOG 0/1, *TP53* change, and *TP53*/*MDM2* change were associated with longer PFS [21]. These results suggest that dysgeusia, which was identified as a prognostic factor in this study, can be easily assessed and may serve as a clinically useful biomarker.

Immunohistochemical staining for Nectin-4 was performed in 169 patients, including 86 patients with muscle-invasive bladder cancer, to investigate the expression of Nectin-4 in each UC subtype [25]. Overall, 50 (58.1%) muscle-invasive tumors were positive for Nectin-4 [25]. Nectin-4 positivity according to histological type was 68.2% in UC, 70% in squamous cell carcinoma, 66% in adenocarcinoma, 28% in patients with micropapillary variants, 50% in those with nested variants, 63% in those with plasmacytoid variants, and 10% in those with sarcomatoid carcinoma [25]. A study evaluating oncologic outcomes in patients with subtypes of UC (SUC) who received EV treatment for la/mUC compared to those with PUC regarding ORR, PFS, and OS has been reported [32]. Although the median PFS was 4.2 months for patients with PUC and 5.9 months for those with SUC (*p* = 0.045), the median OS was not significantly different between the two groups (7.3 months vs. 16.1 months; *p* = 0.065) [33]. However, even among patients with SUC who were evaluated as having CR or PR, the duration of treatment response to EV was significantly shorter compared to patients with PUC (median, 3.7 months vs. 7.3 months, *p* = 0.003) [33]. In this study, we did not examine the SUC in detail because of the small number of patients enrolled; however, we believe that this factor should be examined in the future.

This study has some limitations. First, this was a retrospective study using data from multiple institutions. Therefore, differences in the clinical diagnosis and treatment methods at each institution could have introduced bias in this study. Second, the number of patients enrolled in the study was relatively small and the follow-up period was relatively short. In particular, 28.2% of patients had an ECOG-PS of ≥2 in the EV group compared to 50% of those in the non-EV group, even though the difference was not statistically significant. Third, we did not examine the timing of AEs, such as rash or dysgeusia, after the initiation of EV administration. Fourth, we were unable to investigate the dose and duration of pretreatment medications, as well as the treatment response of EV to different pretreatments. Finally, we could not evaluate effective sequential therapy because of inadequate studies on oncological outcomes after the diagnosis of la/mUC.

## 5. Conclusions

In this multicenter retrospective cohort study, EV treatment appeared to prolong OS in patients with la/mUC who progressed after ICI therapy. NLR <4.18 and the absence of dysgeusia were independent predictors of prolonged OS after the initiation of EV therapy. A large multicenter prospective study is required to validate our results.

## Figures and Tables

**Table 1:** Patient background comparison among three groups.

Covariates	EV Group (n = 64)	Non-EV Group (n = 27)	BSC Group (n = 140)	*p*
Age for starting third-line treatment (median, year, IQR)	72.0 (66.0–77.0)	72.0 (68.0–77.0)	74.0 (70.5–79.0)	0.384
Sex (number, %)				0.686
Male	48 (75.0)	22 (81.5)	103 (73.6)	
Female	16 (25.0)	5 (18.5)	37 (26.4)	
Body mass index (median, kg/m^2^, IQR)	21.0 (19.5–23.4)	21.9 (20.1–24.9)	21.2 (18.7–24.1)	0.532
ECOG-PS (number, %)				
0	21 (32.8)	1 (6.2)	NA	0.126
1	25 (39.1)	7 (43.8)	NA	
2	12 (18.8)	6 (37.5)	NA	
3	5 (7.8)	2 (12.5)	NA	
4	1 (1.6)	0	NA	
Primary tumor site (number, %)				0.930
Bladder	32 (50.0)	16 (59.3)	78 (55.7)	
UUT	23 (35.9)	8 (29.6)	45 (32.1)	
Bladder + UUT	9 (14.1)	3 (11.1)	17 (12.1)	
Histopathology				0.328
pure UC	51 (79.7)	24 (88.9)	104 (74.3)	
UC with any histological subtype	13 (20.3)	3 (11.1)	36 (25.7)	
Surgical resection of the primary site (number, %)	38 (59.4)	18 (66.7)	88 (62.9)	0.790
Patients receiving neoadjuvant therapy (number, %)	22 (50.0)	10 (50.0)	36 (34.3)	0.130
Patients receiving adjuvant therapy (number, %)	12 (27.3)	9 (47.4)	23 (23.2)	0.116
Radiation therapy to the primary site	3 (5.2)	2 (8.0)	9 (6.9)	0.863
Follow-up period after initiation of second-line therapy (months, median)	14.00 (8.0–20.0)	15.00 (6.0–17.5)	6.00 (2.0–14.0)	<0.001
Follow-up period after initiation of third-line therapy (months, median)	6.0 (3.0–11.25)	5.0 (2.5–14.0)	7.0 (7.0–7.0)	0.976
Albumin (g/dL, IQR)	3.6 (3.0–3.8)	3.7 (3.44.1)	NA	0.058
Neutrophil count (/µL, IQR)	4800 (3515–6092)	4000 (2805–5023)	NA	0.083
Lymphocyte count (/µL, IQR)	1037 (723–1379)	1099 (785–1292)	NA	0.738
NLR (IQR)	4.40 (2.86–7.63)	3.32 (2.62–3.96)	NA	0.064
Hemoglobin (g/dL, IQR)	10.8 (9.0–12.4)	11.1 (9.7–12.3)	NA	0.684

EV, enfortumab vedotin; BSC, best supportive care; IQR, interquartile range; ECOG-PS, Eastern Cooperative Oncology Group performance status; UUT, upper urinary tract; UC, urothelial carcinoma; NA, not applicable; NLR, neutrophil-to-lymphocyte ratio.

**Table 2:** Treatment-related adverse events in patients receiving enfortumab vedotin.

Clinical Characteristics	All Grades	Grade ≥ 3
Anemia	22 (34.4)	8 (12.5)
Dysgeusia	20 (31.2)	0 (0.0)
Fatigue	20 (31.2)	0 (0.0)
Nausea	6 (9.4)	0 (0.0)
Decreased neutrophil count	6 (9.4)	5 (7.8)
Peripheral neuropathy	28 (43.8)	0 (0.0)
Rash	25 (39.1)	2 (3.1)

**Table 3:** Uni- and multivariate analysis with OS in patients receiving enfortumab vedotin.

	Univariate			Multivariate		
Variables	HR	95% CI	*p*	HR	95% CI	*p*
Gender (female vs. male)	1.59	0.642–3.954	0.314	1.38	0.514–3.731	0.519
Age at initiation of EV therapy (≥67 vs. 67> years)	3.29	0.990–10.970	0.051	2.72	0.802–9.285	0.108
NLR (>4.18 vs. 4.18>)	3.10	1.340–7.201	0.008	2.50	1.031–6.077	0.042
Dysgeusia (yes vs. no)	0.22	0.082–0.626	0.041	0.30	0.096–0.945	0.039
Rash (yes vs. no)	0.44	0.198–0.992	0.047	1.38	0.514–3.731	0.519

CI, confidence interval; NLR, neutrophil-to-lymphocyte ratio.

## Data Availability

Requests for the data presented in this study should be addressed to the corresponding author. The data are not publicly available for privacy and ethics reasons.
